# Geometry-Dependent Pitch Variation Controls Platelet Adhesion on FluidFM-Fabricated Residual-Layer-Free Micro/Nanostructures

**DOI:** 10.1021/acsomega.5c11820

**Published:** 2026-04-03

**Authors:** Marcus Soter, Dikshita Madkatte, Doris Heinrich, Thi-Huong Nguyen

**Affiliations:** † 84666 Institute for Bioprocessing and Analytical Measurement Techniques (iba), Heilbad Heiligenstadt 37308, Germany; ‡ Institute of Nanotechnology (INT) and Karlsruhe Nano Micro Facility (KNMFi), Karlsruhe Institute of Technology, Karlsruhe 76131, Germany; § Faculty of Mathematics and Natural Sciences, Technische Universität Ilmenau, Ilmenau 98694, Germany

## Abstract

Platelet storage remains a critical challenge in transfusion medicine, with current WHO and FDA guidelines limiting storage to just 72 h due to the risk of platelet dysfunction and bacterial contamination. This study examines how platelet adhesion is influenced by the interstructured distance of various microstructured geometries printed by mask-free nanoimprinting fluid force microscopy (FluidFM) technology. Microstructures of multiple geometries (circles, Pacman, lines, grids, and triangles) were printed on glass surfaces using the commercial Loctite AA3491, composed of multiple acrylate monomers, at three different peak-to-peak distances: 10 μm, 5 μm, and 2 μm. Atomic force microscopy (AFM) was employed to characterize the topography and printing precision of these structures. All structures exhibited nanoscale heights and demonstrated high fidelity to the designed patterns. Adhered platelets on the structured surfaces were quantified using confocal laser scanning microscopy. Results demonstrated that platelet attachment is significantly affected by both structural geometry and peak-to-peak distance. Circular and Pacman-like structures consistently showed reduced platelet adhesion, particularly at the largest peak-to-peak distance of 10 μm. Platelet attachment generally increased with decreasing peak-to-peak distance between the microstructures, yet all structured surfaces showed reduced adhesion compared to unstructured glass and nonpatterned Loctite, indicating that microtopographical modifications can effectively inhibit platelet attachment. Our results provide insights into designing antifouling surfaces for medical applications, demonstrating the potential of FluidFM technology in fabricating precision microstructures to mitigate platelet attachment as a mechanistic model to study geometry-dependent platelet–surface interactions.

## Graphical abstract

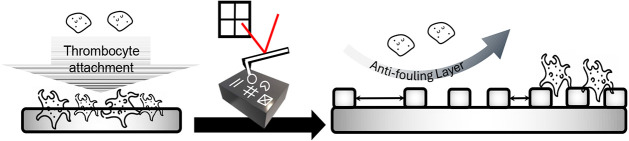

## Introduction

Platelet transfusion therapy represents a critical intervention in modern medicine, yet its clinical efficacy is severely constrained by fundamental storage limitations that have persisted for decades. Current regulatory guidelines from both the World Health Organization (WHO) and the U.S. Food and Drug Administration (FDA) restrict platelet storage to a maximum of 72 h.^1^ This limitation creates significant logistical challenges and potential shortages in clinical settings.^1,2^ This narrow storage window arises from the platelets’ natural tendency to activate and form potentially life-threatening thrombi when they are exposed to artificial plastic surfaces of standard storage containers.^3^

Platelets (plt), also known as thrombocytes, are remarkable cellular fragments with diameters ranging from 1.5 to 3 μm that originate from megakaryocytes in the bone marrow.^4^ Despite comprising less than 1% of the blood volume, platelets are essential elements that play a crucial role in hemostasis and wound healing. Their scarcity in circulation highlights the crucial importance of optimizing storage conditions to maintain their therapeutic efficacy. One of the challenges of platelet storage is their adherence to the synthetic materials of the bags and their subsequent activation. Once adhered, platelets undergo dramatic morphological changes, extending pseudopodia and releasing pro-aggregatory mediators such as adenosine diphosphate (ADP) and thromboxane A2. These chemical signals create a positive feedback loop, recruiting additional platelets and ultimately leading to thrombus formation, which compromises the suitability of stored platelets for transfusion.^5^

Established strategies to mitigate platelet attachment have focused primarily on chemical surface modifications. These approaches involve specific functional groups—such as hydroxyl moieties—to create hydrophilic surfaces that theoretically reduce protein adsorption and subsequent platelet attachment.^6^ While chemically modified surfaces can demonstrate improved biocompatibility in laboratory settings, their clinical implementation faces substantial regulatory hurdles. The medical device approval process requires extensive biocompatibility testing, long-term stability studies, and rigorous evaluation of potential leachable compounds that could contaminate stored blood products.^2^ These requirements translate into prolonged development timelines and substantial financial investments that often prove prohibitive for widespread adoption.

In contrast to chemical modifications, physical surface engineering offers a compelling alternative approach that circumvents many regulatory complications while potentially achieving superior antifouling performance.^7^ Physical modifications alter surface topography without introducing new chemical species, thereby eliminating concerns about leachable compounds while creating surfaces that can actively discourage cellular adhesion through mechanical and geometric effects.

The theoretical foundation for topographical antifouling surfaces draws from well-established principles in surface science, most notably the lotus effect observed in nature.^8^ By creating structured surfaces with controlled micro- and nanoscale features, it becomes possible to manipulate wetting behavior, reduce actual contact area, and create surface states that discourage protein adsorption and cellular adhesion. Critical to this approach is the precise control of structural parameters, particularly the peak-to-peak distance (spacing between features), which governs the transition between different wetting states and directly influences the availability of adhesion sites for platelets.^9^

Recent methods for creating precisely controlled surface topographies, including photolithography, electron beam lithography, and nanoimprint lithography, require complex multistep processes involving master mold fabrication, pattern transfer, and extensive processing steps.^10^ These conventional approaches are not only expensive and time-consuming but also lack the flexibility needed for rapid prototyping and optimization of surface designs.

Fluid Force Microscopy (FluidFM) technology represents a paradigm shift in direct-write surface patterning, combining the precision of atomic force microscopy with the versatility of inkjet printing.^11,12^ This innovative approach employs hollow cantilevers that can deposit precise volumes of material through nanoscale apertures, enabling the direct fabrication of three-dimensional microstructures without the need for masks, templates, or complex lithographic processes. Our previous studies have demonstrated the capability of FluidFM to create structures with heights up to 1 μm and feature sizes in the micrometer range, showing promising results for inhibiting platelet adhesion.^11,12^ However, the optimal peak-to-peak distance of surface structures for maximizing the prevention of platelet adhesion has not yet been systematically evaluated within a residual-layer-free, nanoscale-height regime. While platelet storage bags represent a key long-term application, the present study deliberately employs a simplified, well-controlled model system using washed platelets under static conditions to isolate the effects of surface geometry and peak-to-peak distance on platelet adhesion.

By developing evidence-based design principles for antiadhesive surfaces, storage containers for platelet products will be improved, potentially extending storage times and improving transfusion safety. Also, the principles established here may find broader applications in medical device design, where controlling cellular adhesion is crucial for long-term biocompatibility and device performance.

Recent studies have demonstrated that micro- and nanoscale surface patterning can modulate platelet adhesion by controlling feature geometry and peak-to-peak distances. In particular, FluidFM-fabricated nanopatterns with acrylate-based resins and hydrogel surfaces have shown that circular features and large peak-to-peak distances can reduce platelet attachment, while edge-rich geometries tend to promote adhesion.^11,13^ However, these earlier investigations primarily focused on relatively tall microstructures or soft hydrogel substrates, where platelet responses may be influenced by residual polymer layers, bulk material properties, or protein-mediated effects.^14^ We have previously found that characteristics of nanostructures, including height, width, and cross-line, depend on several technical issues, such as the printing direction and structural features.^11,12^ However, it remains unclear how peak-to-peak distances influence platelet adhesion when topographical features are confined to the sub-100 nm height regime. In this work, we independently vary peak-to-peak distance (2, 5, 10 μm) among structures, including circles, Pacmans, lines, grids, and triangles, fabricated by mask-free FluidFM printing utilizing Loctite AA3491 ink. The study is a fully systematic geometry–spacing analysis within one material system, allowing mechanistic identification of spacing limits in the prevention of platelet-surface adhesion. By combining residual-layer-free nanostructuring with quantitative platelet adhesion analysis, this study establishes geometry- and spacing-dependent design rules that extend beyond earlier FluidFM-based patterning approaches and provide mechanistic insight into spacing-driven platelet inhibition on stiff glass surfaces.

Our results reveal clear structure-dependent trends in platelet attachment, with specific round geometries and larger peak-to-peak distances significantly reducing adhesion. These findings not only advance our understanding of how microstructured surfaces modulate cellular responses but also offer practical design principles for antifouling strategies in biomedical devices, particularly for enhancing the safety and performance of platelet storage systems and other blood-contacting materials.

This investigation represents a contribution step toward solving one of transfusion medicine’s most persistent challenges while advancing our understanding of how precisely engineered surface topographies can control biological responses at the cellular level.

## Methods

### Ethical Considerations and Blood Collection

Human blood obtained from healthy volunteers, including the informed consent procedure, was approved by the ethics board at the Thüringen Körperschaft des öffentlichen Rechts, Landesärztekammer Thüringen, 07707 Jena, Germany (Number: 38627/2023/37). All volunteers gave informed consent.

### Substrate Preparation and Spin Coating

To apply a uniform layer of the polymer on the surfaces, 300 μL of Loctite AA3491 (Henkel, Düsseldorf, Germany), was applied on glass coverslip surfaces (Plano GmbH, Wetzlar, Germany), and spun with a WS-500B 400BZ-6NPP Lite spin coater (Laurell Technologies Corporation, Pennsylvania) at 3000 rpm for 60 s, and cured for 5 min using a 365 nm UV lamp.

### FluidFM-Based Microstructure Fabrication

Microstructures were printed using an AFM Nanowizard 4 system (Bruker, Berlin, Germany) equipped with a FluidFM add-on (Cytosurge, Opfikon, Switzerland) and a hollow cantilever (MAT FluidFM Nanosyringe, 300 nm, 0.6 N/m). The structures were designed using Inkscape (version 0.17) as vector scale graphics and loaded in the AFM control software JPK Nanowizard 4 (Bruker, Berlin, Germany). While printing, the movement of the cantilever was observed using the inverted microscope Axio Observer (Zeiss, Jena, Germany). As printing material, the commercially available Loctite AA3491 (Henkel, Düsseldorf, Germany) was used.^11^ The imprinting resin consists of monomers from isobornyl acrylate, 2-hydroxyethyl methacrylate, acrylic acid, and hydroxypropyl acrylate, which can be cured using UV light.^15^ The cantilever was calibrated in a contact-free thermal noise method before printing. The structures were printed on a glass coverslip (Plano GmbH, Wetzlar, Germany) using the manipulation mode. The printing was done using a set point of 20 nN, a positive pressure of 20 mbar, and a writing speed of 20 μm/s. The printed features were cured using a 365 nm UV lamp for 5 min.

### Surface Characterization by Atomic Force Microscopy

For characterization of the printed structures, the AFM Nanowizard 4 (Bruker, Berlin, Germany) was used. As a cantilever, the MLCT (A) (tip radius of ∼20 nm, nominal spring constant of 0.07 N/m) (Bruker, Berlin, Germany) was used. The scan size was set to 100 × 100 μm^2^. All samples were imaged using a line rate of 0.3 Hz and a resolution of 512 × 512 pixels in contact mode. The images were processed, and the images and line profile analysis was obtained, using the JPK Nanowizard software (Bruker, Berlin, Germany). Height, width, and peak-to-peak distance measurements were analyzed and presented as the mean ± standard deviation of at least 15 measurements for three independently fabricated samples per condition.^11^

### Platelet Isolation and Preparation

All platelet experiments were conducted using blood from three healthy voluntary donors who had not consumed any medication/drugs in the previous 2 weeks. Whole blood was collected into 10 mL tubes prefilled with 1.5 mL acid–citrate–dextrose (ACD-A) (BD Vacutainer, Germany). Immediately after collection, tubes were sealed (parafilm), inclined at ∼45°, and rested at room temperature for 15 min before processing. Platelet-rich plasma (PRP) was obtained by centrifugation at 120 g for 20 min at room temperature. Platelets were isolated from PRP by centrifugation at 650 g for 7 min in the presence of 11% ACD-A (Fresenius Kabi, Germany) and 2.5 U/mL apyrase (grade IV, Sigma, Germany). The platelet pellet was gently resuspended in 1 mL suspension buffer (pH 6.3) containing 137 mM NaCl, 2.7 mM KCl, 11.9 mM NaHCO_3_, 0.4 mM Na_2_HPO_4_, and 2.5 U/mL hirudin, after which 4 mL of the same buffer was added and the suspension was incubated for 15 min at 37 °C. Platelets were then centrifuged again at 650 g for 7 min and finally resuspended in 2 mL suspension buffer. Platelet concentration was determined using an automated blood counter (pocH-100i, Sysmex, Germany) and subsequently adjusted to a low concentration of 50,000 platelets/μL to promote formation of a monolayer of single platelets on control glass and structured substrates, enabling reliable platelet counting and spreading-area assessment. Before experiments, platelets were allowed to rest for 45 min at 37 °C. To minimize preactivation, all handling steps were performed using gentle pipetting (no vortexing; low shear), and platelet isolation included ACD-A, apyrase, hirudin, and a pH 6.3 suspension buffer. Preactivation was monitored on the pocH-100i by checking the platelet size distribution (and absence of large-particle/aggregate signals) before applying platelets to surfaces; samples showing signs of abnormal size distribution/aggregation were excluded.^8,11,12^

### Platelet Adhesion Analysis by Confocal Microscopy

For observing platelet attachment on the investigated surfaces, laser scanning microscopy was used. Therefore, after incubation of the platelets for 15 min on the desired structures, the platelets were fixed using Histofix (Carl Roth GmbH& Co. KG, Karlsruhe, Germany) and stained with anti-CD42a Alexa Fluor 488 antibodies 0.1 μg/mL (Dinova GmbH, Hamburg, Germany) at RT for 30 min before being washed with PBS twice to remove all the unbound antibodies. The samples are observed using a Zeiss LSM 710 (Carl Zeiss, Jena, Germany) confocal microscope. The excitation wavelength was set to 495 nm, and emission was collected at 550 nm using a 63× water-immersed objective. Platelets were isolated from 3 independent healthy donors. For each condition (geometry × peak-to-peak distance), platelets were incubated on three independently fabricated surfaces (technical replicates), and the complete surface of 100 × 100 μm^2^ was imaged. Platelet counts and sizes were quantified in ImageJ (ImageJ 1.54d, Wayne Rasband and contributors, National Institutes of Health, USA). Platelet spreading area was quantified in ImageJ using a standardized particle-analysis workflow. Confocal images were background-corrected and thresholded identically across conditions, followed by binarization and Analyze Particles to extract the projected platelet area. Objects were filtered by size (2–30 μm^2^) and circularity (0–1) to exclude noise and platelet aggregates, and the resulting segmentation was visually verified on representative images before exporting area values for statistical analysis. For statistical analysis, platelet count and the average size of all attached platelets across the whole surface of one condition were taken. Technical replicates were averaged per donor before inferential statistics.^11^

### Surface Properties Characterization by Contact Angle Measurements

For measuring the contact angle of the glass substrate (Plano GmbH, Wetzlar, Germany) and the Loctite resin (AA3491, Henkel, Düsseldorf, Germany), the OCA 15+ system (Data-Physics Instruments GmbH, Filderstadt, Germany) was used. Three μL of Milli-Q water (0.055 μS/cm) was applied on the substrates and fitted by the polynomial fitting in the sessile drop method. The calculation was done by OCA 15+ software (Data-Physics Instruments GmbH, Filderstadt, Germany).^8^ Each value is the mean of three measurements on three independent surfaces.

### Surface Properties Characterization by Zeta Potential Measurements

The Anton Paar SurPASS 3 (Anton Paar GmbH, Graz, Austria) was used to measure the zeta potential, and the data were recorded using the SurPASS 3 software (Anton Paar GmbH, Graz, Austria). The SurPASS 3 uses the streaming potential technique, where the potential difference generated by the streaming flow was measured.^16^ Loctite was applied by spin coating on the glass surface as described before (section Substrate Preparation and Spin Coating). The UV-cured sample was mounted in the measuring cell. The sample was placed between two reservoirs, which are a cleaning chamber and an electrolyte reservoir filled with a 1 M NaCl, 7 pH, 25 °C solution. For the measurement, a pressure gradient of 200–600 mbar and a gap height of 100–120 μm were used. Three independently prepared samples per condition were measured in 3 zeta cycles, and the chamber was cleaned after each measurement.

### Single Molecule Force Spectroscopy (SMFS)

To determine the adhesion of platelets to the surfaces, single-molecular force spectroscopy (SMFS) experiments were performed. The FluidFM cantilever with an aperture of 800 nm and 2 N/m spring constant (Cytosurge, Opfikon, Switzerland) was used and attached to the AFM (Nanowizard, Bruker, Berlin, Germany). The nozzle of the cantilever was brought into contact with a platelet, and a negative force of −800 mbar was applied to pick up the platelet. After the pick-up, the negative pressure was reduced to −200 mbar, and the cantilever with the attached platelet was removed from the surface. Afterward, in force-mapping mode, on a 20 × 20 μm^2^ area on the spin-coated samples, 50 force curves per sample were acquired before changing the platelet on the cantilever. Three different platelets per spin-coated sample were picked up for measurements. In the force mapping mode, a set point of 5 nN, a z-length of 5 μm, a pulling speed of 2 μm/s, and an attachment time of 3 s to allow the platelets to get in contact with the surface.^12^ Data processing was done using the JPKSPM data processing software, and the adhesion force was calculated accordingly. The mean and standard deviation of three different platelets per donor (*n* = 3) on the different surfaces were calculated.

### Statistical Analysis

Data are shown as mean ± SD unless stated otherwise. Mean and standard deviation were calculated using Origin, Version 2023b (OriginLab Corporation, Northampton, MA, USA). Platelet adhesion was analyzed using a two-way repeated-measures ANOVA with the factors geometry and peak-to-peak distance (including interaction). Multiple comparisons were corrected using Tukey posthoc testing. Significance was set at α = 0.05 (****p < 0.0001; ***p < 0.001; **p < 0.01; *p < 0.05; ns > 0.05) using GraphPad Prism 8.0 (GraphPad Software, San Diego, California, USA).

## Results

To systematically investigate platelet adhesion behavior across varying surface topographies, five distinct geometric patterns (circle, Pacman, line, square, and triangle) were designed at three different peak-to-peak distances (10-, 5-, and 2 μm) using Inkscape (Figure 1). In case of the round geometries (circle, Pacman), the peak-to-peak distance describes the diameter of a single feature and the interstructure distance, which are equal, while for the linear geometries (line, grid), the peak-to-peak distance describes the distance between two lines. For the triangle structures, the peak-to-peak distance describes the altitude of the triangle (see red arrows in Figure 1). These patterns were strategically selected to examine how different topographical features influence platelet attachment mechanisms through distinct surface characteristics that affect platelet-surface response. Circular and Pacman patterns provide curved structures that may promote different platelet spreading dynamics compared to the angular features of square and triangle patterns. Platelets typically exhibit enhanced adhesion on curved surfaces due to better conformational matching with their natural morphology.^11,12^ While the circular structures show no edges, Pacman structures show one, triangles three, and squares four edges, where platelets might adhere.^11^ The varying peak-to-peak distances create different edge densities, with smaller peak-to-peak distances providing more frequent topographical transitions. Since platelets preferentially adhere to surface edges, higher edge densities are expected to increase available adhesion sites and enhance overall platelet attachment.^17^ Additionally, the fabrication process introduces systematic variations in surface topography through overlapping print paths of square, and triangle structures that create distinct microenvironments for platelet interaction.

**Figure 1:**
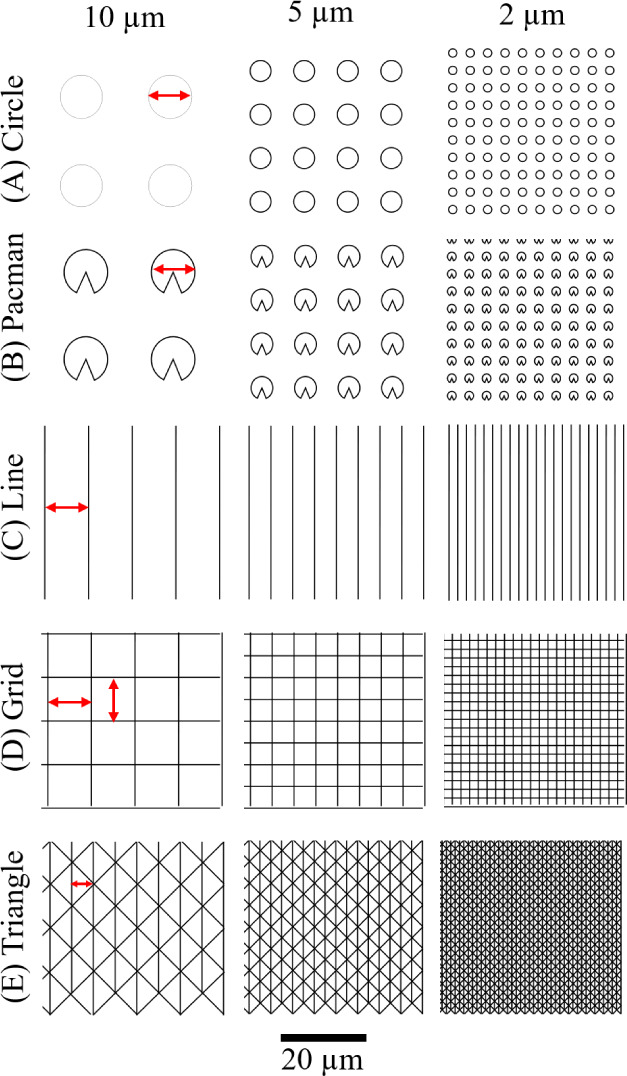
Design of the surface geometries, including circle (A), Pacman (B), line (C), squares (D), and triangle (E), with peak-to-peak distances of 10-, 5-, and 2 μm. Red arrows indicate the diameters.

These overlap-induced height differences create microscale surface roughness that may enhance platelet attachment through increased contact area and mechanical stimulation. Line patterns exhibit no overlap regions, creating uniform height profiles, while square patterns show single overlaps at intersections that generate localized height variations. Triangle patterns demonstrate the most complex topography with double overlaps at junction points, producing the most pronounced topographical variations.

### Dimensional Accuracy and Fabrication Fidelity

All patterns were fabricated on glass substrates using FluidFM cantilever technology to ensure consistent baseline surface properties across all printed structures, allowing for direct comparison of pattern-specific effects on platelet adhesion behavior. To validate the printing accuracy of FluidFM lithography using Loctite AA3491 and assessing the resulting topographical features, a comprehensive dimensional analysis of AFM images (Figure 2) of the fabricated surface patterns was performed. The characterization revealed distinct microstructural peak-to-peak distances (10-, 5-, and 2 μm), and all geometric configurations were quantified in Table 1. The measured data points, as illustrated in Figure 2A, confirm that the FluidFM fabrication process successfully reproduced the designed geometries with sufficient precision to enable meaningful comparative analysis of platelet behavior. The comparison between designed peak-to-peak distance and measured peak-to-peak distance values (Table 1) demonstrated an excellent fabrication fidelity, with measured dimensions closely matching intended specifications across all pattern types. This dimensional accuracy is crucial for establishing reliable structure–function relationships in platelet adhesion studies because minor deviations in feature size can significantly impact cellular response mechanisms. Importantly, the microstructural analysis revealed that while maintaining consistent peak-to-peak distance, each geometric pattern creates distinct local surface environments that may present different mechanical and topographical cues for platelets.

**Figure 2:**
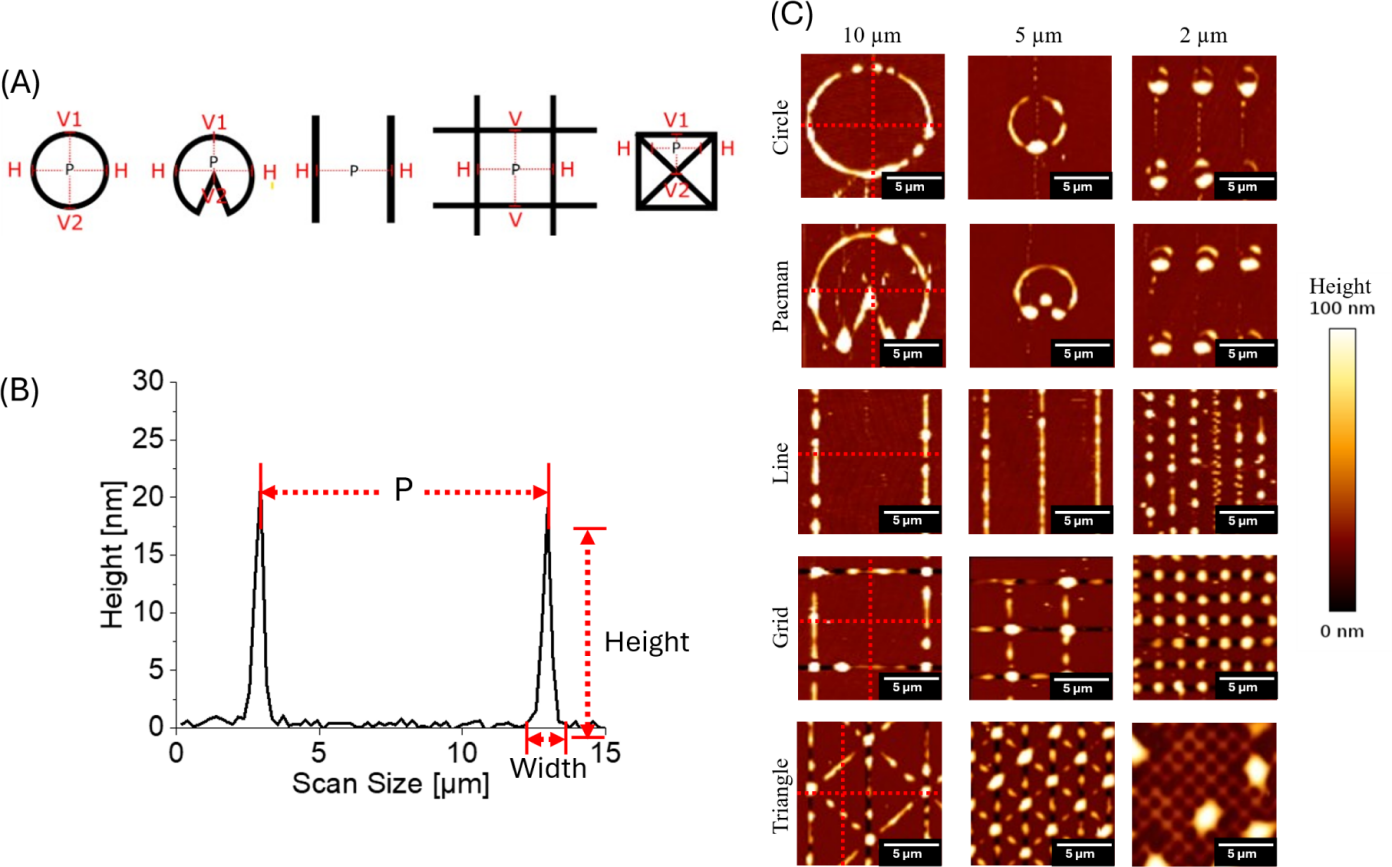
(A) Schematic representation of the AFM measurement points for different printed structures from left to right: circle, Pacman, line, grid, and triangle structures. Red labels indicate measurement positions: H (horizontal), V1 (first vertical), V2 (second vertical), and P-2-P (peak-to-peak distance). Dotted red lines show the exact measurement trajectories used to determine the dimensional characteristics of each geometry. (B) Representative AFM line profile of line structure for the determination of peak-to-peak distance, height, and width (red arrows). Peak-to-peak distance (C) AFM topography images of different structures (rows) printed with varying peak-to-peak distances (columns). Structures include circles, Pacman-like shapes, lines, grids, and triangles, fabricated with 10-, 5-, and 2 μm peak-to-peak distance. The color bar represents height variations, with brighter areas indicating higher structures. All AFM height images are displayed with an identical z-range (0–100 nm). Red lines indicate, where the quantitative profiles were taken.

**Table 1:** Dimensional Analysis of Different Printed Structures Measured by AFM^a^

		**Peak-to-peak distance**								
		10 μm			5 μm			2 μm		
		Height [nm]	Width [μm]	Peak-to-peak distance [μm]	Height [nm]	Width [μm]	Peak-to-peak distance [μm]	Height [nm]	Width [μm]	Peak-to-peak distance [μm]
Circle	H	22 ± 6	1.021 ± 0.137	10.127 ± 0.383	24 ± 8	1.096 ± 0.135	5.009 ± 0.272	21 ± 5	0.978 ± 0.142	2.011 ± 0.102
	V-1	22 ± 8	0.803 ± 0.137	9.790 ± 0.075	25 ± 11	0.965 ± 0.126	4.817 ± 0.079	29 ± 10	0.930 ± 0.099	1.873 ± 0.072
	V-2	43 ± 20	1.510 ± 0.193		110 ± 31	1.649 ± 0.157		104 ± 11	1.475 ± 0.138	
Pacman	H	30 ± 9	1.090 ± 0.199	10.055 ± 0.103	40 ± 16	1.207 ± 0.207	5.045 ± 0.144	29 ± 11	0.779 ± 0.118	1.800 ± 0.127
	V-1	16 ± 4	1.129 ± 0.116	6.042 ± 0.101	17 ± 7	1.170 ± 0.127	3.297 ± 0.097	33 ± 12	1.226 ± 0.135	1.570 ± 0.095
	V-2	82 ± 12	2.375 ± 0.265		94 ± 9	1.866 ± 0.152		114 ± 9	0.779 ± 0.118	
Line	H	23 ± 9	1.351 ± 0.196	10.001 ± 0.75	15 ±5	1.204 ± 0.146	5.001 ± 0.078	15 ± 8	1.073 ± 0.155	2.012 ± 0.103
Grid	H	18 ± 6	1.315 ± 0.136	9.962 ± 0.78	28 ± 11	1.318 ± 0.137	5.007 ± 0.091	23 ± 6	1.109 ± 0.094	2.031 ± 0.114
	V	17 ± 5	1.111 ± 0.136	10.031 ± 0.067	21 ± 7	1.066 ± 0.115	5.036 ± 0.095	25 ± 9	1.023 ± 0.127	1.978 ± 0.086
Triangle	H	31 ± 11	1.572 ± 0.177	4.938 ± 0.326	35 ± 11	1.443 ± 0.151	2.532 ± 0.178	Not measurable		
	V-1	22 ± 10	1.152 ± 0.134	1.767 ± 0.185	47 ± 12	1.249 ± 0.210	2.542 ± 0.118			
	V-2	98 ± 17	1.767 ± 0.185		92 ± 17	1.651 ± 0.120				

a The structures (circle, Pacman, line, grid, and triangle) were printed with varying peak-to-peak distances (10-, 5-, and 2 μm). Height, width, and peak-to-peak distance measurements are presented as the mean of at least 15 measurements per three independently fabricated samples per condition ± standard deviation. Peak-to-peak distance values represent the actual distance between adjacent structures. V-1 and V-2 measurements correspond to distinct positions within the same structure, with V-2 typically showing larger dimensions due to the merging effect of adjacent structures. “Not measurable” indicates where structures could not be reliably quantified due to dimensional constraints at the smallest peak-to-peak distance.

The measured peak-to-peak distances provide insights into printing fidelity and are defined as the distance between the peaks of two neighboring features.

For a designed 10 μm peak-to-peak distance, the measured peak-to-peak distance values showed remarkable accuracy across all structures. The grid structure demonstrated high fidelity with measurements of 9.962 ± 0.078 μm (horizontal peak-to-peak distance) and 10.031 ± 0.067 μm (vertical peak-to-peak distance). Similar precision was observed in circles (9.790 ± 0.075 μm ), Pacman structures (10.055 ± 0.103 μm), and lines (10.001 ± 0.075 μm). Triangular structures showed a peak-to-peak distance value of 4.938 ± 0.326 μm as designed. The base of the triangle was designed with a length of 10 μm, resulting in a peak-to-peak distance value of the altitude of 5 μm between the first vertical, V1, and the second vertical, V2 (Figure 2A). Therefore, there is a high degree of precision between the design and the measured value for all structure types.

At a designed 5 μm peak-to-peak distance, the measurements again showed a strong correlation with the intended design. Grid structures maintained high accuracy with peak-to-peak distance values of 5.007 ± 0.091 μm (horizontal) and 5.036 ± 0.095 μm (vertical). Lines (5.001 ± 0.078 μm), circles (4.817 ± 0.079 μm), and Pacman structures (5.045 ± 0.144 μm) all exhibited values within ∼5% of the designed spacing. Triangular structures are also in line with the design, measuring 2.532 ± 0.178 μm. For the smallest 2 μm peak-to-peak distance, slight deviations from the designed spacing became more pronounced, although they still maintained reasonable accuracy. Measured values ranged from 1.873 ± 0.072 μm for circles to 2.031 ± 0.114 μm for grids’ horizontal peak-to-peak distance. Notably, triangle structures became unmeasurable at this spacing. Here, the designed peak-to-peak distance between peak distance between V-1 and V-2 are only 1 μm. The combination of the resin’s flow behavior and the substrate’s wetting behavior results in the merging of the two lines. Therefore, it is not possible to measure the peak-to-peak distance here. For circular structures, the vertical measurements showed two distinct height regions. The first vertical measurement (V-1) exhibited consistent heights across all peak-to-peak distances (22 ± 8 nm, 25 ± 11 nm, and 29 ± 10 nm for 10-, 5-, and 2 μm peak-to-peak distances, respectively). However, the second vertical measurement (V-2) demonstrated significantly larger heights, ranging from 43 ± 20 nm at 10 μm peak-to-peak distance to 104 ± 11 nm at 2 μm peak-to-peak distance. These variations in height occur due to the openings of the cantilever, which have a straight and a half-circle side as described previously.^11^ This leads to an unequal amount of ink being carried, depending on the printing direction. Pacman structures displayed similar dimensional characteristics, with V-2 measurements consistently showing the largest heights (82 ± 12 nm, 94 ± 9 nm, and 114 ± 9 nm for decreasing peak-to-peak distance). The H measurements remained stable across different peak-to-peak distances, averaging around 30–40 nm in height. Linear structures demonstrated the most consistent height measurements, with H-heights ranging from 23 ± 9 nm at 10 μm peak-to-peak distance to 15 ± 8 nm at 2 μm peak-to-peak distance. Grid structures showed similar consistency in both H and V directions, with heights typically between 17–28 nm across all peak-to-peak distances. Triangle structures showed unique behavior, becoming unmeasurable at a 2 μm peak-to-peak distance due to the merging of two adjacent lines. At larger spacings, they exhibited significant height differences between V-1 and V-2 measurements, with V-2 heights reaching 98 ± 17 nm at 10 μm peak-to-peak distance, which can be attributed to the overlapped printing.

The data demonstrate that most structures can be printed with peak-to-peak distances closely matching the designed peak-to-peak distance at 10 and 5 μm, with precision slightly decreasing at 2 μm spacing. The triangle structures showed the largest deviations in height and became unmeasurable at the smallest peak-to-peak distance.

### Peak-to-Peak Distance and Structure-Dependent Platelet Adhesion Behavior

To investigate the response of platelet adhesion to different shapes and peak-to-peak distances, the platelet solution of 50,000 plt/μL was incubated on the printed structures for 15 min on the surface. After fixing and staining them with anti-CD42a Alexa Fluor 488, a platelet-specific antibody, the surfaces were imaged by using confocal microscopy (Figure 3A). To quantify platelet adhesion, the number of adhered cells within the whole surface area was compared among structured surfaces. Platelet adhesion/spreading was quantified for 3 independent donors (*n* = 3). For each donor and condition, three independently fabricated surfaces were analyzed (technical replicates; total of 9 surfaces per condition). Platelet adhesion on all structured surfaces was significantly reduced compared to that of the nonpatterned Loctite surface or bare glass (Figure 3B). At a peak-to-peak distance of 10 μm, shape-dependent prevention of platelet adhesion was observed to be the strongest (Figure 3B). Here, for the circle structures, no bound platelets were observed on the surface. On the Pacman (2.6 ± 1.2 plt/10^4^ μm^2^) and triangular structures (2.6 ± 2.49 plt/10^4^ μm^2^), a slight increase in bound platelets was found. On a more edge-like structure, an increased number of platelets was found, with platelet counts of 11.0 ± 2.5 plt/10^4^ μm^2^ on grids and 9.0 ± 2.9 plt/10^4^ μm^2^ on lines. With a decrease in the peak-to-peak distance for these structures, an increase in the amount of bound platelets was observed. At a peak-to-peak distance of 5 μm, the circular structures show the lowest amount (2.3 ± 0.9 plt/10^4^ μm^2^) of bound platelets. For the circular-like structure, the amount of platelets is decreased with Pacman (4.6 ± 1.7 plt/10^4^ μm^2^) and triangular shapes (3.3 ± 3.2 plt/10^4^ μm) compared to lines (7.0 ± 3.5 plt/10^4^ μm^2^) and grids (8.0 ± 2.1 plt/10^4^ μm^2^). For a peak-to-peak distance of 2 μm, the differences between the structure geometries in the amount of attached platelets became minor. The lowest amount of platelets (4.6 ± 4.0 plt/10^4^ μm^2^) was observed for the triangles, while the other geometries showed an equal amount of bound platelets (8.6 ± 1.8 plt/10^4^ μm^2^ for Pacman, 9.3 ± 0.5 plt/10^4^ μm^2^ for circles, 10.0 ± 3.7 for lines, and 10.6 ± 5.7 plt/10^4^ μm^2^ for grids). In contrast to the glass control (15.0 ± 2.1 plt/10^4^ μm^2^), all structured surfaces showed a reduction in the amount of bound platelets. The reduction is prominent, in contrast to the unstructured Loctite (63.0 ± 8.2 plt/10^4^ μm^2^).

**Figure 3:**
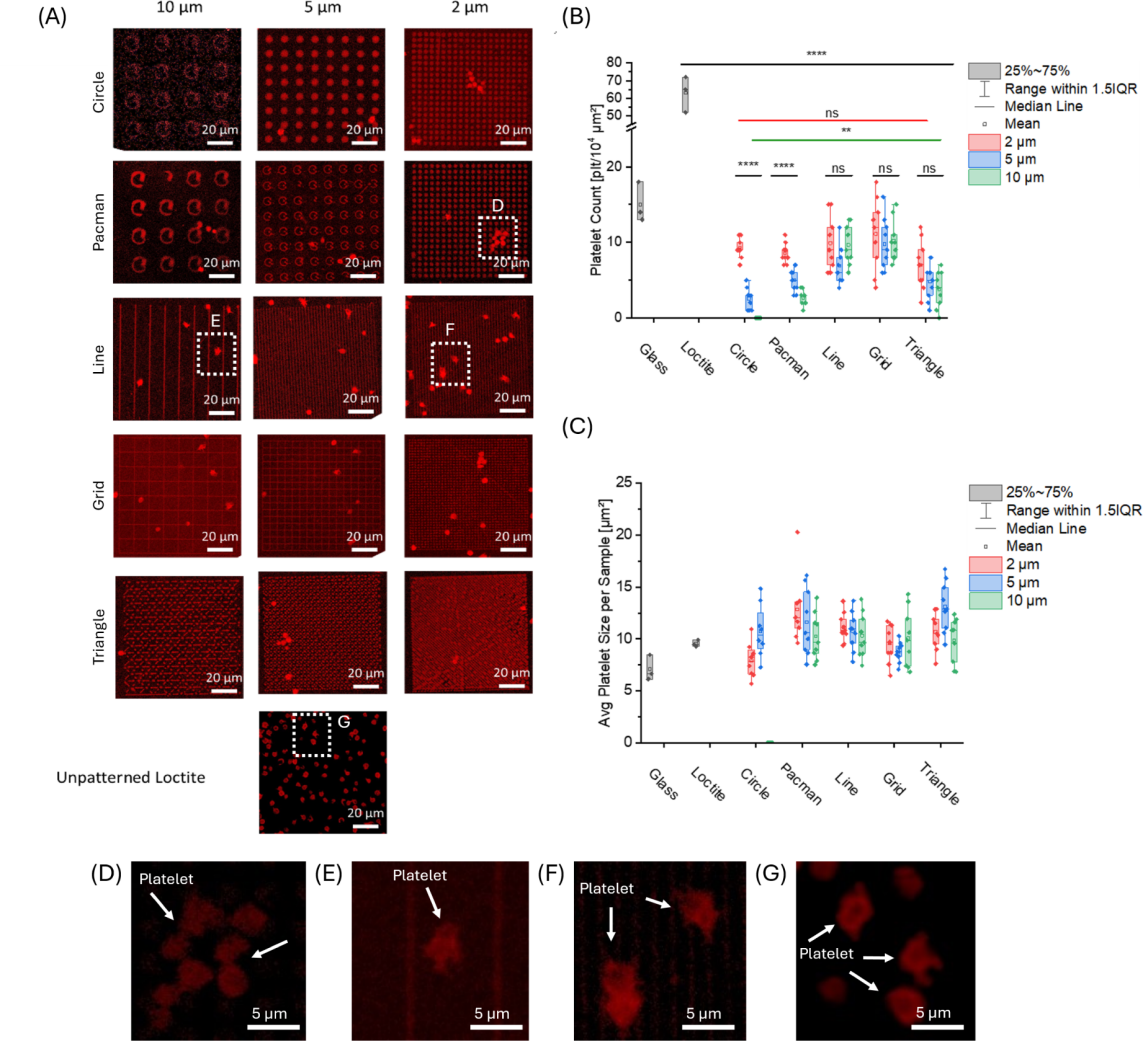
Quantification of platelets attached to the different structures and peak-to-peak distances. (A) Confocal scanning microscopy images of adhered platelets stained with anti-CD42a Alexa Fluor 488 in red on the differently shaped and with different peak-to-peak distances printed, structures. (B) Analysis of platelet-bound surface; with a decrease in the peak-to-peak distance, an increase in the platelet attachment is observed. Overall, circular structures like circles and Pacman show a reduction in platelet attachment. For quantification, platelets from three independent donors were analyzed; for each donor and condition, three independently fabricated surfaces were imaged (technical replicates) and averaged per donor. (C) Analysis of the average platelet size per structure and peak-to-peak size. Statistical analysis by two-way repeated-measures ANOVA with the factors geometry and peak-to-peak distance (including interaction). Statistics were performed on donor-level averages (*n* = 3 donors); each donor mean is based on three technical replicate surfaces. Multiple comparisons were corrected using Tukey posthoc testing. Significance was set at α = 0.05 (****p < 0.0001; ***p < 0.001; **p < 0.01; *p < 0.05; ns > 0.05). (D–G) Representative higher-magnification confocal images of adherent platelets taken from the dashed regions indicated in (A): (D) Pacman geometry, 2 μm peak-to-peak distance; (E) Line geometry, 10 μm peak-to-peak distance; (F) Line geometry, 2 μm peak-to-peak distance; (G) unpatterned Loctite control surface. White arrows indicate representative platelets.

On flat reference surfaces, platelets exhibited a mean spreading area (Figure 3C) of 7.10 ± 1.23 μm^2^ on glass and 9.57 ± 0.32 μm^2^ on Loctite. In contrast, microstructured surfaces showed geometry-specific, pitch-dependent changes in spreading (Figure 4C). For circle patterns, the average spreading area increased from 7.92 ± 1.69 μm^2^ at 2 μm pitch to 10.76 ± 2.53 μm^2^ at 5 μm, whereas no quantifiable spreading was observed at 10 μm pitch, consistent with no detectable adherent platelets on circle (10 μm) under our imaging/analysis conditions (values plotted as 0). Pacman structures yielded the largest spreading at 2 μm (12.85 ± 3.15 μm^2^), with a decrease at larger pitches (11.63 ± 3.21 μm^2^ at 5 μm and 10.26 ± 2.24 μm^2^ at 10 μm). Line patterns were comparatively pitch-insensitive, with mean spreading areas around 10–11 μm^2^ across pitches (11.12 ± 1.39 μm^2^, 10.76 ± 1.86 μm^2^, and 10.32 ± 2.14 μm^2^ for 2-, 5-, and 10 μm). For grid patterns, spreading was lowest at 5 μm (8.75 ± 0.99 μm^2^) compared with 2 μm (9.56 ± 1.84 μm^2^) and 10 μm (10.08 ± 2.77 μm^2^). Finally, triangle patterns showed maximal spreading at 5 μm (13.11 ± 2.47 μm^2^), while 2 μm (10.69 ± 1.78 μm^2^) and 10 μm (9.86 ± 2.16 μm^2^) resulted in lower areas. The boxplots with overlaid individual data points highlight the variability within each condition and support a geometry- and pitch-dependent modulation of platelet spreading.

**Figure 4:**
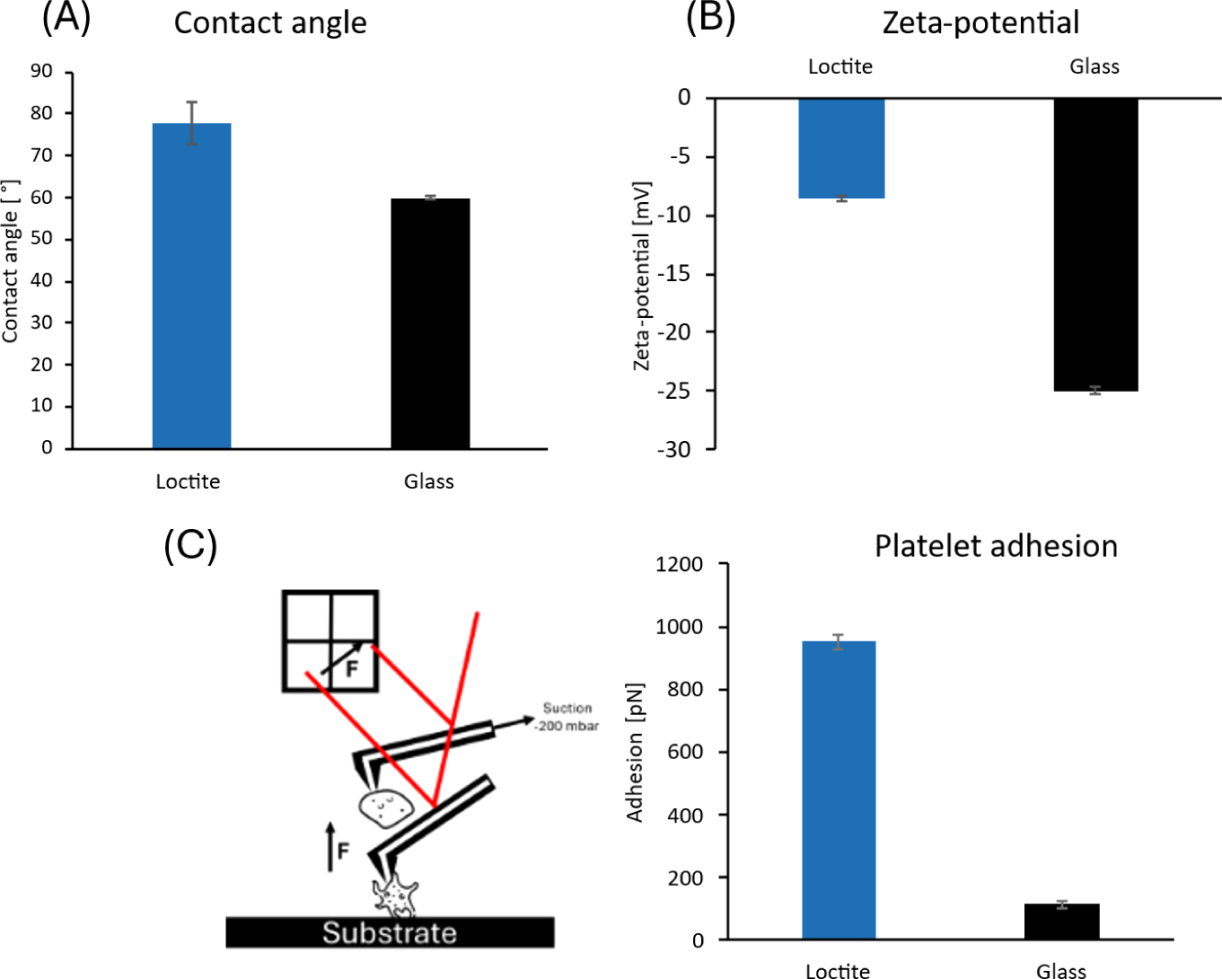
Characterization of nonpatterned Loctite surface compared to glass for the effect of platelet attachment. In contrast to glass, the water contact angle (A) and the platelet adhesion force measured by SMFS on Loctite (C) are increased. The zeta potential (B) on Loctite surface is less negative than on glass. For the contact angle and zeta potential, 3 repeats on three different samples were performed, and the mean and standard deviation are shown. In SMFS measurements, 50 force curves per platelet, on three different samples per surface, were analyzed to calculate the mean and standard deviation.

### Surface and Bio Interface Characterization of Loctite Film

It is known that physical surface characteristics can influence the adhesion of proteins, bacteria, and platelets. To understand the mechanism that governs the changes in platelet-surface adhesion, the contact angle, zeta potential, and platelet-surface adhesion force of nonstructured Loctite AA 3491 films compared to glass were analyzed. The water contact angle of Loctite-coated surfaces (77.9° ± 5.0°) is increased as compared with glass (60.5° ± 1.7°) (Figure 4A). The zeta potential of Loctite (−8.6 ± 0.2 mV) is less negative than that of glass (−25 ± 0.3 mV) (Figure 4B). Here, the electrostatic potential of the surface is measured. Both parameters were previously reported to be in correlation with the adhesion of platelets.^17,18^ A decreased contact angle and zeta potential provide an increased antifouling capability. This can be seen well when observing the adhesion force (Figure 4C) measured using the FluidFM. By applying negative pressure to the microchannel, a platelet is sucked into the cavity of the FluidFM cantilever. With the attached platelet, the cantilever is brought into contact with the surface, and the detachment force is measured. The adhesion of the platelets on Loctite (951 ± 24 pN) is higher than that on glass (114 ± 13 pN).

## Discussion

FluidFM offers a versatile and precise approach for fabricating micro- and nanostructures directly onto surfaces, enabling customized patterning without the need for complex and cost-intensive processes such as silicon mold production, etching, or photolithographic development. In this study, we employed FluidFM to create antifouling surfaces specifically designed to reduce platelet adhesion- a critical consideration for blood-contacting biomedical devices. To evaluate the impact of surface geometry on platelet interactions, we systematically investigated the effect of peak-to-peak distance (10-, 5-, and 2 μm) across different structural features. Our results show that platelet attachment is significantly governed by surface geometry and peak-to-peak distance, with circular and Pacman-like structures, especially at 10 μm peak-to-peak distance, exhibiting the lowest platelet adhesion. Compared to unstructured glass and nonpatterned Loctite surfaces, all structured patterns reduced platelet attachment, indicating that micro- to nanoscale spatial organization can effectively inhibit adhesion.

The dimensional analysis across different geometries and peak-to-peak distances reveals several additional insights into the capabilities and constraints of FluidFM nanofabrication technology. Our results demonstrate significant improvements in printing precision and dimensional control compared to previous work, while also identifying new limitations at smaller scales.^11^ The peak-to-peak distances achieved in this study show exceptional accuracy, with deviations of less than 2% from designed values for larger spacings. Grid structures demonstrated the highest precision, achieving 9.962 ± 0.078 μm for a 10 μm designed spacing and 5.007 ± 0.091 μm for 5 μm spacing. This represents a substantial improvement in dimensional fidelity compared to earlier FluidFM applications, where dimensional variations were more pronounced.^11,12^ Notably, the changed printing parameters in comparison to our previous work (increased printing speed and reduced pressure) enabled the fabrication of structures with significantly reduced heights (22–114 nm) while maintaining structural integrity and geometric fidelity.^11^

Similar trends have been observed in other studies examining nano- and microstructured surfaces with varying peak-to-peak distances and sizes, even tested with different cell types. For instance, it has been demonstrated that nanoscale features with heights in the range of 50–200 nm can influence cellular adhesion and behavior in fibroblasts and epithelial cells, highlighting the importance of finely tuned dimensions for directing cell-material interactions.^19,20^ Furthermore, investigations into neuronal cells revealed that even sub-100 nm height topographies can modulate neurite outgrowth and alignment in contrast to micrometer height topographies, suggesting that these dimensions are functionally relevant across diverse biological systems.^21^ These findings underscore the potential of our refined nanometer-scaled height printing approach to fabricate biologically relevant structures.^22,23^

A critical finding of this study is the identification of geometry-specific printing limitations. Triangle structures cannot be differentiated at a 2 μm peak-to-peak distance due to resin merging between adjacent features, revealing a fundamental constraint related to the interplay between designed geometry, feature spacing, and resin flow dynamics. This limitation is particularly pronounced when the designed peak-to-peak distance approaches the resin spreading diameter, suggesting a minimum feature separation threshold of 1.5–2 μm for complex angular geometries.

It was shown that the edges in structures lead to increased resin deposition due to the cantilever’s momentary pause before changing direction. This effect becomes particularly problematic at smaller peak-to-peak distances, explaining our observations. Due to the design of the triangular structures, the base of the triangle was designed with a distance of 2 μm; therefore, the altitude (peak-to-peak distance) value becomes only 1 μm. Here, the peak-to-peak distance becomes too small, leading to the merging of the resin. This also presents the limitations of the FluidFM printing. Here, the interplay of resin spreading and writing speed seems to be the critical factor. A higher viscosity of resin would lead to less spreading and, therefore, open the possibility of smaller structures. The upper limit for resin viscosity is determined by the system’s maximum applicable pressure and the flow resistanc e induced by the narrow nozzle diameter. The directional dependence observed in height measurements (V-1: 22–33 nm vs V-2: 43–114 nm for circular structures) confirms the asymmetric deposition pattern characteristic of FluidFM printing. The variation in structure dimensions across different geometries and printing directions observed in our study can be attributed to similar mechanisms as those described previously.^11,12^ The authors reported that printing direction significantly influences structural dimensions due to the asymmetric geometry of the FluidFM probe tip, featuring a straight opening on one side and a semicircular opening on the other.^11,12^ The results presented here also indicate this directional dependence particularly clearly in the height measurements, where two distinct height regions can be observed in circular structures (V-1: 22–29 nm; V-2: 43–104 nm) and Pacman structures (V-1: 16–33 nm; V-2: 82–114 nm). However, our systematic analysis across multiple geometries confirms the findings by Soter et al., that this directional bias can be leveraged predictively to achieve desired structural asymmetries, potentially opening innovative design possibilities for applications requiring controlled anisotropic surface features.^11^

Our results reveal a clear relationship between feature size and printing accuracy. Even the precise peak-to-peak distance, particularly at larger spacings (e.g., 9.962 ± 0.078 μm for 10 μm designed spacing in grid structures), demonstrates good dimensional control. This improvement is due to optimized printing parameters. While structures at 10- and 5 μm peak-to-peak distances showed excellent dimensional control, deviations became more pronounced at 2 μm spacing, with an accuracy of 93–94% of designed values. This scale-dependent behavior suggests that the current FluidFM parameters approach a resolution limit around 2 μm for reliable multifeature printing. The different printing parameters, particularly the relationship between pressure and writing velocity, were identified as crucial factors affecting structure dimensions and precise printing.^12^ However, the structures here generally showed lower heights compared to those reported by Soter et al. (with printed heights of 220–818 nm, depending on the structure).^11^ Our optimized parameters produced more uniform, lower-profile structures (15–114 nm) with superior dimensional consistency. This trade-off between height and precision represents a significant advancement for applications requiring precise surface patterning with minimal vertical relief.

The viscosity-dependent spreading behavior observed, particularly in triangular structures at small peak-to-peak distances, highlights the critical role of resin rheology in determining achievable feature densities. Our findings suggest that current resin formulations impose fundamental limitations on minimum feature spacing, independent of cantilever precision. This represents a key area for future material development, as higher viscosity resins could enable smaller peak-to-peak distances but would require correspondingly higher printing pressures. The width consistency achieved across different structures (0.8–2.4 μm) demonstrates improved process control compared to earlier reports, suggesting that optimized printing parameters can effectively minimize feature-to-feature variations while maintaining throughput efficiency.

Our findings regarding the relationship between designed peak-to-peak distances and actual peak-to-peak distances provide new insights. The high accuracy achieved at larger spacings (10- and 5 μm) and the slight deviations at 2 μm spacing suggest that while the FluidFM technique is highly precise for features >2 μm, there may be fundamental limitations when approaching smaller dimensions, due to the resin spreading mechanisms. These findings contribute to design rules for FluidFM-based surface patterning applications. The demonstrated precision at micrometer scales, combined with the identified limitations at submicrometer spacings, provides clear guidance for optimizing pattern design based on intended applications. The ability to achieve reliable sub-100 nm height while maintaining micrometer-scale lateral precision positions FluidFM as a viable alternative to traditional lithographic techniques for specific applications requiring moderate resolution with high design flexibility. These results complement existing knowledge about FluidFM printing capabilities and suggest that optimizing printing parameters based on structure geometry and intended spacing might be crucial for achieving the desired dimensional accuracy, particularly at smaller scales.

Quantitative validation that the fabricated structures maintain their designed spacing, ensuring that observed differences in platelet adhesion can be attributed to geometric pattern effects rather than dimensional variations. This characterization establishes the foundation for interpreting subsequent biological responses, as the precise control of surface topography enables direct correlation between specific geometric features and platelet attachment mechanisms.

The amount of bound platelets strongly depended on the peak-to-peak distance, with smaller distances leading to increased adhesion. Fallon et al. reported similar effects in linear PVA structures under static incubation conditions, consistent with the findings of the present study.^24^

A direct quantitative comparison with earlier FluidFM- and nanoimprint-lithography (NIL)-fabricated antifouling surfaces reveals both strong continuity and important distinctions in the present results. Early FluidFM approaches reported by Apte et al. showed platelet adhesion levels of approximately 20–60 plt/10^4^ μm^2^ for hive- and dot-like Loctite structures with structure heights below 50 nm, indicating partial inhibition relative to unstructured polymer surfaces.^12^ Subsequent FluidFM-based nanopatterning on Loctite by Soter et al., employing structure heights in the range of 200–800 nm, demonstrated that circular and Pacman-like geometries consistently reduced platelet adhesion relative to edge-rich patterns such as grids or lines, particularly at larger interstructural spacings. In those studies, circular features typically supported 2–6 plt/10^4^ μm^2^, whereas grid- and line-based patterns exhibited higher adhesion levels of 8–15 plt/10^4^ μm^2^, depending on spacing.^11^ Similar geometry-dependent trends were reported for agarose-based NIL micro- and nanostructures, where round patterns reduced platelet adhesion by approximately 40–70% compared to grid-like features, corresponding to adhesion levels in the range of 2–17 plt/10^4^ μm^2^ in platelet storage models.^8^

The present study confirms and extends these trends under markedly different structural conditions. Despite a substantially reduced structure height compared to Soter et al. of only 15–114 nm and the absence of a residual polymer layer, circular structures at a peak-to-peak distance of 10 μm exhibited no detectable platelet adhesion, while Pacman-like and triangular geometries showed similarly low adhesion levels of 2–3 plt/10^4^ μm^2^.^11^ In contrast, edge-rich geometries such as grids and lines displayed higher adhesion of 9–11 plt/10^4^ μm^2^ under identical conditions. Notably, decreasing the peak-to-peak distance from 10 to 2 μm led to a consistent increase in platelet adhesion across all geometries, with adhesion levels converging toward 9–11 plt/10^4^ μm^2^, comparable to or exceeding values previously reported for significantly taller FluidFM-fabricated structures. Compared to earlier work, these findings demonstrate that effective platelet inhibition, particularly for rounded geometries, can be achieved at nanoscale heights, while reduced structure height generally lowers platelet adhesion for both dot- and grid-like patterns. Together, these comparisons show that the relative geometric ranking of antifouling performance is preserved across fabrication methods, materials, and height regimes, while emphasizing that edge density and peak-to-peak distance remain dominant parameters governing platelet–surface interactions.

Beyond platelet counts, the platelet spreading area was calculated to assess how microtopography affects postadhesion activation. The spreading-area data indicate that geometry and pitch modulate not only the number of adherent platelets, but also the extent of platelet activation/spreading once adhesion occurs. Notably, the absence of detectable platelets on circle patterns at 10 μm pitch suggests that this geometry–pitch combination falls below a threshold required for stable initial attachment, potentially due to reduced feature density and fewer effective anchoring sites. In contrast, edge-rich/asymmetric motifs (e.g., Pacman) or locally confined contact regions (e.g., triangle at 5 μm) support larger spreading areas, consistent with stronger outside-in signaling on these topographies. The comparatively pitch-insensitive response on Line patterns suggests that continuous ridge-like cues provide persistent contact guidance even when spacing changes, supporting a geometry- and pitch-dependent coupling between adhesion and spreading. Overall, these trends support a geometry- and pitch-dependent “adhesion-to-activation” coupling, which should be further validated by combining spreading metrics with activation markers (e.g., CD62P/PAC-1) in future work.

Koh et al. demonstrated that submicrometer surface structuring effectively reduces platelet attachment, whereas micrometer-scale structuring exhibits no significant influence on adhesion.^25^ Their investigation examined platelet adhesion on pillar structures fabricated from poly­(lactic-*co*-glycolic acid). In contrast to their findings, the results presented in this study demonstrate that increased peak-to-peak distances lead to decreased platelet attachment. It is likely that not only structures, but also platelet-contacting materials played a role. While platelets in our study contact directly with the polymerized acrylates (Loctite), their structured surfaces were preincubated with fibrinogen before platelet exposure. It is known that fibrinogen reduces the aggregation and adhesion of platelets.^26,27^ Since the protein adsorption is sensitive to peak-to-peak distances, the effects observed in their study likely reflect alterations in fibrinogen adsorption rather than the effect of the peak-to-peak distances. Consequently, their findings primarily indicate how structural dimensions modulate protein distribution and subsequent platelet-protein interactions, whereas the current study investigates direct platelet adhesion to uncoated surfaces. Similar effects must be considered when examining the findings of Bui et al., who demonstrated slightly reduced platelet adhesion forces on laminin-coated nanostructured surfaces, although the number of adhered platelets was not quantified in their study.^13^ The protein coating efficiency is strongly dependent on surface interspacing, resulting in significant variations in coating homogeneity that consequently influence platelet adhesion behavior. This effect is particularly pronounced when coating sub-100 nm structures with large proteins such as laminin, where protein deposition can effectively reduce the available surface and thereby diminish the intended nanostructuring as seen in surface characterization. This phenomenon was not clearly observed by Pham et al., who investigated platelet attachment on various topographies of poly­(dimethylsiloxane) (PDMS).^28^ The experiments conducted by Pham et al. were under flow conditions, whereas the present study was performed under static conditions. Under static conditions, surface wettability properties exert a more pronounced influence on platelet behavior compared to flow conditions. Additionally, since the material properties of PDMS differ significantly from those of Loctite, the distinct material characteristics of Loctite must be considered when interpreting these results. It is known that higher wettability leads to a reduction in the adhesion of proteins and cells. The attached water molecules can form a water barrier and hinder the proteins from attaching.^29^ Here, the contact angle on glass is lower than on Loctite, leading to a decreased amount of attached platelets. When the feature size and the peak-to-peak distance decrease, more Loctite resin is deposited on the surface, meaning the structured area increasingly exhibits the material properties of Loctite rather than glass. As a result, decreasing the peak-to-peak distance led to an increase in platelet adhesion.

Besides the water barrier, the zeta potential can be taken into consideration. It is well-known that a more negative zeta potential hinders platelet attachment.^30,31^ The zeta potential of Loctite is less negative compared to that of glass. It is known that the surface of platelets, which consists of negatively charged glycocalyx, is repulsed from more negative surfaces. Moreover, the activation path includes the passive agglutination and activation of integrin αIIbβ3 on these more positively charged surfaces.^30^ Therefore, a more negative zeta potential increases the likelihood of platelet repulsion. Together, these parameters explain why platelet binding forces to Loctite are higher than to glass. Consistently, Apte et al. reported greater platelet adhesion on unpatterned Loctite surfaces compared to glass, whereas structured Loctite surfaces, such as hives and dot-like (grid) structures, showed reduced attachment.^12^ In addition, the adhered platelets on unpatterned Loctite showed reduced spreading, which was associated with the lower stiffness of Loctite compared to glass. However, because FluidFM structuring produces features of limited height without a Loctite residual layer, platelets establish broader contact with the underlying glass substrate than with the resin. Consequently, the influence of Loctite on platelet spreading can be considered negligible (Figure 4, Grid 10 μm).

SMFS measurements revealed that platelet adhesion forces on unpatterned Loctite were nine times higher than on glass, indicating a strong affinity of platelets for the resin surface. However, at a peak-to-peak distance of 10 μm, the number of adhered platelets decreased. This reduction appears to be influenced not by material properties but by structural effects such as geometry. Structures with rounded features exhibited fewer attached platelets, consistent with our previous observations, despite differences in structural height.^11^ In the present study, the lower structures bound fewer platelets compared to the taller structures reported previously,^11^ likely because increased height provides a larger resin surface area for platelet attachment, whereas reduced height limits adherence. Besides these effects, the topographical change in the surfaces reduces platelet attachment. It is well established in the literature that an increased surface roughness increases the amount of adhered platelets, whereas a systematic pattern in the micrometer range seems to reduce the adhesion.^32^ This phenomenon can be attributed to topography-induced variations in platelet attachment mechanisms between the two materials (structured resin and substrate), which subsequently alter the platelet attachment behavior for each surface. In our study, even unsystematic patterns like the triangle 2 μm structures lead to a reduction in the platelet attachment compared to the line and grid structures, while the effect of contacting materials of both glass and Loctite can also play a role. Nevertheless, a round geometry like circles seems to reduce the attachment most efficiently, mimicking the natural tissue, which leads to no attached platelets on these structures. It should be noted that the present experiments were performed under deliberately simplified conditions using washed platelets at low concentration on glass-supported Loctite structures under static incubation. This reductionist approach was chosen to decouple geometric and spacing effects from confounding factors such as plasma proteins, shear forces, leukocytes, and bacterial contamination that are present in platelet concentrates stored in polymer bags under agitation. While these conditions differ substantially from clinical storage environments, they enable a mechanistic assessment of how surface geometry and peak-to-peak distance alone influence platelet adhesion. Future studies will need to evaluate whether the design principles identified here are preserved under more complex and clinically relevant storage conditions, including platelet-rich plasma, longer incubation times, flow or agitation, and storage-bag polymers such as PVC. Further tests should be the investigation, that the structures can be transferred to other imprint techniques, allowing larger areas and highly cost-effective imprints, to further investigate the behavior of the round surface structures on platelets, closer to clinical conditions. Adhesion alone may not fully capture thrombogenic potential because platelets may adhere while remaining relatively quiescent or conversely exhibit activation pathways that are not reflected by counts. Accordingly, future work will extend the present geometry-controlled platform by integrating activation-specific end points (e.g., CD62P/P-selectin expression, PAC-1 binding, phosphatidylserine exposure) and coagulation-related assays (e.g., fibrin deposition and thrombin generation, ideally under flow) to directly link early adhesion phenomena to downstream thrombo-inflammatory responses.

## Conclusions

The FluidFM technique demonstrates significant potential as a precise and flexible platform for fabricating structured surfaces with tailored antifouling properties for medical applications. This study reveals a mechanistic model for the relationship between microstructure geometry, peak-to-peak distances, nanoscale height, and platelet adhesion, factors that are critical in the design of blood-contacting materials. Unlike traditional lithographic approaches, FluidFM allows for high-fidelity patterning with minimal processing steps, representing a useful advance in surface engineering for medical use. Our results show a complex, peak-to-peak distance-dependent behavior of platelet adhesion, with increased platelet attachment observed as the spacing between features decreases. Notably, circular and Pacman-like structures consistently inhibited platelet adhesion, underscoring the importance of topographical cues in modulating cellular response. These findings highlight the potential of microstructured surfaces to regulate platelet interactions and pave the way for more effective antifouling strategies in medical devices, particularly in applications such as platelet storage systems. By advancing our understanding of how microstructure design influences platelet-surface interactions, this contributes to the mechanistic framework for the design of antifouling surface topographies in blood-contacting materials aimed at reducing platelet attachment and thrombus formation.

## Data Availability

All data generated or analyzed during this study are included in this published article file. Raw data and additional materials are available from the corresponding author upon reasonable request.
